# Frequency of visits to Tomioka town and related factors among evacuees more than a decade after the Fukushima Daiichi Nuclear Power Plant accident

**DOI:** 10.1093/jrr/rrad018

**Published:** 2023-04-06

**Authors:** Hitomi Matsunaga, Xu Xiao, Varsha Hande, Makiko Orita, Yuya Kashiwazaki, Yasuyuki Taira, Noboru Takamura

**Affiliations:** Department of Global Health, Medicine and Welfare, Atomic Bomb Disease Institute, Nagasaki University, 1-12-4 Sakamoto, Nagasaki 852-8523, Japan; Department of Global Health, Medicine and Welfare, Atomic Bomb Disease Institute, Nagasaki University, 1-12-4 Sakamoto, Nagasaki 852-8523, Japan; Department of Global Health, Medicine and Welfare, Atomic Bomb Disease Institute, Nagasaki University, 1-12-4 Sakamoto, Nagasaki 852-8523, Japan; Department of Global Health, Medicine and Welfare, Atomic Bomb Disease Institute, Nagasaki University, 1-12-4 Sakamoto, Nagasaki 852-8523, Japan; Department of Global Health, Medicine and Welfare, Atomic Bomb Disease Institute, Nagasaki University, 1-12-4 Sakamoto, Nagasaki 852-8523, Japan; Department of Global Health, Medicine and Welfare, Atomic Bomb Disease Institute, Nagasaki University, 1-12-4 Sakamoto, Nagasaki 852-8523, Japan; Department of Global Health, Medicine and Welfare, Atomic Bomb Disease Institute, Nagasaki University, 1-12-4 Sakamoto, Nagasaki 852-8523, Japan

**Keywords:** Fukushima Daiichi Nuclear Power Plant accident, frequency of visits, intention to return

## Abstract

This study aimed to clarify the frequency of visits (FOV) to Tomioka town, Japan, and related factors among evacuees more than a decade after the Fukushima Daiichi Nuclear Power Plant accident. A questionnaire survey was conducted on residents (age ≥ 18 years) who had residence cards in August 2021. Of the 2260 respondents, the FOV to Tomioka was as follows: 926 (41.0%) more than twice a year (Group 1 [G1]), 841 (37.2%) once a year (G2) and 493 (21.8%) no visits (G3). About 70% of the respondents who had decided not to return to Tomioka visited once a year or more. No significant differences in the FOV or radiation risk perception were found between groups. Multinomial logistic regression analysis using G3 as a reference revealed independent associations between living inside Fukushima in G1 (odds ratio [OR] = 5.4, 95% confidence interval [CI]: 4.1–7.3; *P* < 0.01) and G2 (OR = 2.3, 95% CI: 1.8–3.0, *P* < 0.01), undecided about returning in G1 (OR = 2.5, 95% CI: 1.9–3.3, *P* < 0.01), females in G1 (OR = 2.0, 95% CI: 1.6–2.6, *P* < 0.01) and motivation to learn more about tritiated water in G2 (OR = 1.8, 95% CI: 1.3–2.4, *P* < 0.01). Overall, 80% of the residents had visited Tomioka within a decade after the accident. These findings suggest the need to continue the effective dissemination of information about the effects of a nuclear accident and the subsequent decommissioning process to evacuees after evacuation orders have been lifted.

## INTRODUCTION

In March 2011, an accident occurred at Tokyo Electric Power Company’s Fukushima Daiichi Nuclear Power Plant (FDNPP) as a result of the Great East Japan Earthquake and resulting massive tsunami [1]. The earthquake and consequent tsunami caused widespread devastation and the loss of nearly 20 000 lives. Although not fully confirmed in multidisciplinary research, no loss of life has been attributed to the various radionuclides released from the FDNPP [2]. However, 164 865 people were evacuated because of the FDNPP accident: 102 827 inside and 62 038 outside of Fukushima. At the beginning of the aftermath of the FDNPP accident, decisions regarding the need for evacuation and sheltering were made based on the current plant conditions [3]. The massive tsunami that occurred immediately after the earthquake led to the loss of reactor core cooling, three nuclear meltdowns and three hydrogen explosions. However, a few months after the accident, better solutions were found for various problems, including the handling of the cooling system at the plant, so designations regarding evacuation orders shifted depending on the estimated annual cumulative doses of radiation in three areas to avoid unnecessary radiation exposure: ‘less than or equal to 20 mSv (evacuation order cancellation preparation zone),’ ‘may exceed 20 mSv but less than 50 mSv (restricted residence zone)’ and ‘exceeding 50 mSv (difficult to return zone)’ [1, 3]. The total area under evacuation orders issued on 23 April 2011 was up to 12% (~1.792 km^2^/13.783 km^2^) of the whole area of Fukushima Prefecture. From 10 March 2020, evacuation orders were gradually lifted in 2.7% (371 km^2^/13.783 km^2^) of the prefecture based on decreases in estimated annual cumulative doses [4]. In August 2021, the Japanese government announced a policy for lifting evacuation orders that defined special zones for reconstruction and revitalization. In doing so, residents were allowed to enter a limited area (~28 km^2^) after decontamination, even in the ‘difficult to return’ zone [5, 6] Although the lifting of evacuation orders around the FDNPP area was widely promoted, a total of 33 360 people (6481 inside and 22 727 outside of Fukushima) were continuing to live as evacuees as of August 2022 [7]. Return rates differed vastly between municipalities. For example, the return rate for Kawauchi village, to which evacuees started returning in 2012, exceeded 80%, whereas that for Futaba town was still only a few percent in 2022 [8, 9].

The earthquake (seismic intensity with a magnitude above 6), tsunami (height about 21.1 m) and nuclear accident on 11 March 2011 severely damaged Tomioka town, which is located within 10–20 km of the FDNPP. Immediately after the disaster, all the residents of Tomioka had to evacuate after the evacuation order was issued [10]. Many residents evacuated to neighboring Kawauchi village before moving on to Koriyama, Fukushima and Iwaki cities, which are all more than ~50 km away from Tomioka, whereas others had to evacuate to other areas on their own [10, 11].

On 1 April 2017, at 6 years after the nuclear accident, the Japanese government declared that the radiation air dose rates were at acceptable levels, and thus the residents who had previously resided in ~88% of Tomioka could return to their homes [5]. However, on 1 April 2022, just 5 years after the evacuation order had been lifted, the return rate was still only 9.6% (1816/11 947) [11].

Regardless of this low return rate, to our knowledge, no study has been conducted to clarify residents’ frequency of visits (FOV) to Tomioka and related factors. The trend in residents’ intention to return (ITR) to Tomioka has not changed dramatically since the evacuation order was lifted, with about 60% of residents decided not to return, 25% undecided and 15% wanted to return [12]. Previous studies have already revealed that ITR is related to factors such as sociodemographic characteristics, risk perception and anxiety about radiation exposure [13–16]. FOV could also be related to factors such as sociodemographic characteristics, risk perception and anxiety about radiation exposure, similar to the relationships with ITR, as well as other factors that have not been clarified. Residents who have high anxiety about radiation exposure and radiation risk perception may have lower FOV than those who do not.

In April 2021, the Japanese government and Tokyo Electronic Power Company decided to release Advanced Liquid Processing System-treated water that contained trace amounts of radionuclides (hereinafter tritiated water) from the FDNPP into the Pacific Ocean [17]. Although this is essential for the reconstruction progress after the FDNPP accident, there are substantial safety concerns such as the effects of radiation on health and the environment and reputational damage to industries such as fisheries [18]. Tomioka town has an important seaport for industry that began operating as soon as the evacuation order was lifted [19]. Therefore, the residents of Tomioka may have higher motivation to learn about tritiated water and acquire more basic radiation knowledge. Clarifying the characteristics of individuals interested in tritiated water could be expected to be useful for reconstruction efforts in Fukushima. Given this background, to clarify how to provide adequate support to evacuees more than a decade after the FDNPP accident, this study aimed to clarify the evacuees’ FOV and related factors, including demographic characteristics, ITR, radiation risk perceptions and recognition of information and knowledge about radiation.

## METHODS

### Study participants

**Fig. 1:**
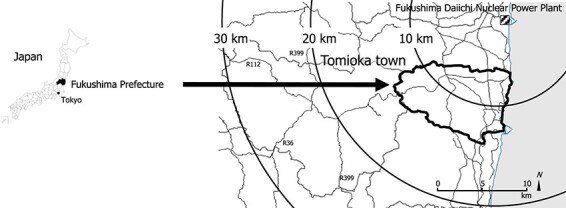
Location of Tomioka town and FDNPP in Fukushima Prefecture, Japan.

The present survey was conducted in November 2021 and January 2022, a little over a decade after the FDNPP accident. The study region was Tomioka town in Fukushima Prefecture, close to the FDNPP (Fig. 1). We distributed questionnaires by post to 9655 Tomioka residents aged >18 years who had residence cards as of 11 March 2011, and still had them as of August 2021. Of 2899 total responses (response rate: 30.1%), 2546 were regarded as valid after excluding incomplete responses (valid response rate: 87.8%). Data were obtained from 286 (11.2%) residents who had already returned to Tomioka, 1442 (56.6%) who had decided not to return, 258 (10.1%) who wanted to return and 560 (22.0%) who were still undecided. As the aim of this study was to clarify the FOV to Tomioka and related factors among the residents who had not returned, the data from the 286 residents who had already returned were excluded from analysis. Permission to conduct this study was obtained from the municipal government of Tomioka, based on a partnership agreement regarding risk communication such as radiation anxiety [20]. The questionnaire was prepared at the university and then distributed to Tomioka town from the Radiation Health Management Section using the Basic Resident Ledger. We explained the study purpose, methods and ethical considerations using a leaflet, and responding to the questionnaire was considered to indicate informed consent. All processes in this study were reviewed and approved by the Nagasaki University Graduate School of Biomedical Sciences Ethics Committee (No. 21082702) in accordance with the Declaration of Helsinki.

### Data collection

The questionnaire was developed based on our previous studies conducted in the area of Fukushima Prefecture where evacuation orders had already been lifted [14, 21]. Those studies surveyed risk perception regarding radiation exposure and its health effects and included a lifestyle survey conducted within the framework of the Fukushima Health Management Survey, organized by Fukushima Prefecture [22, 23].

Furthermore, we added questions about tritiated water and basic knowledge regarding radiation, which were considered of high interest to the residents of Tomioka. The self-administered questionnaires used in this study asked the residents how frequently they had visited Tomioka from October 2020 to November 2021 within the most recent year at the time they responded. The possible responses were: ‘several times a week (146; 6.5%),’ ‘several times a month (329; 14.6%),’ ‘several times in a half year (451; 20.0%),’ ‘once a year (841; 37.1%)’ and ‘not in the last year (493; 21.8%).’ Accordingly, to clarify the relationship between the FOV and factors such as radiation risk perception and ITR, the respondents who had visited Tomioka more than twice a year were defined as Group 1 (G1), once a year as Group 2 (G2) and not at all as Group 3 (G3). Although there were other classification options for the FOV, the G1, G2 and G3 classifications were adapted to ensure a proportional balance for the respondents and enable a better understanding of the interpretation of the study results.

We also classified the respondents based on their responses regarding their ITR to Tomioka as follows: had already returned, had decided not to return, wanted to return and undecided. We also asked the residents about issues related to radiation, such as whether they were reluctant to consume food (e.g. mushrooms, wild plants) from Tomioka, whether they thought adverse health effects were likely to occur as a result of the FDNPP accident, whether they thought genetic effects were likely to occur as a result of the FDNPP accident and whether they had considered consulting a professional in regard to radiation. Furthermore, we asked whether they were motivated to gain more basic knowledge about radiation and tritiated water. The responses to these questions were: ‘yes,’ ‘probably yes,’ ‘probably no’ and ‘no.’ We classified responses of ‘yes’ and ‘probably yes’ as ‘yes,’ and responses of ‘no’ and ‘probably no’ as ‘no.’ We also asked (‘yes’ or ‘no’) whether the residents had knowledge of a free food inspection center in Tomioka, a free personal dosimeter rental system for residents and a place that offered consultations about radiation. The following demographic data were collected from all respondents: sex, age, employment, living with a child (age < 18 years) and area of residence at the time of the survey (inside or outside of Fukushima). We then classified the respondents into age groups as follows: 20 s (88; 3.5%), 30 s (96; 3.8%), 40 s (217; 8.5%), 50 s (360; 14.1%), 60 s (688; 27.0%), 70 s (716; 28.1%) and ≥80 years (381; 15.0%). Finally, we consolidated the age categories as <60 and ≥60 years.

### Statistical analyses

The Chi-square test was used to investigate differences in factors depending on the FOV to Tomioka. First, we compared G1 and G2 to confirm whether the perceptions of each factor differed depending on the FOV to Tomioka, such as once a year or more. Second, to confirm the differences between visitors and non-visitors, we also compared G1 + G2 vs G3 using the Chi-square test. These Chi-square tests were performed separately and independently. Then, factors that independently differed among groups were further investigated using multinomial logistic regression analysis with G3 as a reference. The explanatory variables used in the calculation for the multinomial logistic regression analysis were sex, age, current area of residence, ITR, motivation to gain more basic knowledge about radiation and tritiated water and having knowledge of a place that offers consultations about radiation, which showed a significant difference in G1 + G2 vs G3.

Before performing the multinomial logistic regression analysis, to exclude the possibility of collinearity, it was confirmed that the correlation coefficient (*r*) was <0.8. The respondents knew about the free food inspection center in Tomioka and free personal dosimeter rental system was eliminated from the model calculations. In the multinomial logistic regression analysis, ITR was divided into ‘I want to return and others’ and ‘Not decided yet and others’ based on the distribution of responses. The results of the model fit and likelihood ratio tests were both <0.001, indicating the good fit of the model. Furthermore, the variance inflation factor adapted from the model was also <5. *P*-values < 0.05 were considered significant. All statistical analyses were performed using SPSS version 28.0.1.0 (142) (IBM, Armonk, NY, USA).

## RESULTS

Of the 2260 residents (not including the residents who had already returned), the FOV to Tomioka was as follows: 926 (41.0%) more than twice a year (G1), 841 (37.2%) once a year (G2) and 493 (21.8%) no visits (G3). Table 1 lists the sociodemographic factors and results of the Chi-square test for all groups. Compared with G2, G1 had a significantly higher percentage of females (G1 vs G2; 57.3 vs 42.1%, *P* < 0.001), those who were employed (G1 vs G2; 36.4 vs 25.0%, *P* < 0.001), those who lived inside Fukushima (G1 vs G2; 89.5 vs 79.0%, *P* < 0.001), those who had decided not to return to Tomioka (G1 vs G2; 17.3 vs 8.0%, *P* < 0.001), those who were still undecided about returning to Tomioka (G1 vs G2; 31.1 vs 21.4%, *P* < 0.001), those who wanted to return to Tomioka (G1 vs G2; 51.6 vs 70.6%, *P* < 0.001), those who knew about the free food inspection center in Tomioka (G1 vs G2; 74.4 vs 63.4%, *P* < 0.001) and those who knew about the free personal dosimeter rental system for residents (G1 vs G2; 55.5 vs 50.5%, *P* = 0.040). No significant differences in age, living with a child, reluctance to consume food from Tomioka, concern that adverse health effects and genetic effects would occur because of the FDNPP accident, considering consulting a professional in regard to radiation, motivation to learn more basic knowledge about radiation and treated water, and knowing about a place that offers consultations on radiation were found between G1 and G2.

**Table 1:** Sociodemographic characteristics of the participants in relation to the FOV to Tomioka town

	Response	Overall % (*N*) 100 (2260)	G1 % (*n*) 41.0 (926)	G2 % (*n*) 37.2 (841)	*P*-value^a^	G3 % (*n*) 21.8 (493)	*P*-value^b^
Sex	Male Female	51.9 (1174) 48.1 (1086)	42.7 (395) 57.3 (531)	57.9 (487) 42.1 (354)	<0.001	59.2 (292) 40.8 (201)	<0.001
Age (years)	<60 ≥60	30.6 (692) 69.4 (1568)	28.9 (268) 71.1 (29.1)	27.7 (233) 72.3 (608)	0.301	38.7 (191) 61.3 (302)	<0.001
Employment	Yes No	30.8 (695) 69.2 (1565)	36.4 (337) 63.6 (589)	25.0 (210) 75.0 (631)	<0.001	30.0 (148) 70.0 (545)	0.367
Living with a child (age < 18 years)	Yes No	16.9 (381) 83.1 (1879)	18.3 (169) 81.7 (33.5)	15.9 (134) 84.1 (707)	0.110	15.8 (78) 84.2 (415)	0.267
Current area of residence	Inside Fukushima Outside Fukushima	80.0 (1809) 20.0 (451)	89.5 (829) 10.5 (97)	79.0 (664) 21.0 (177)	<0.001	64.1 (316) 35.9 (177)	<0.001
ITR	No ITR Undecided Want to return	63.8 (1442) 24.8 (560) 11.4 (258)	17.3 (160) 31.1 (288) 51.6 (478)	8.0 (67) 21.4 (180) 70.6 (594)	<0.001	6.3 (31) 18.6 (92) 75.1 (370)	<0.001
Reluctant to consume food from Tomioka	Yes Probably Yes Probably no No	24.3 (549) 28.4 (642) 31.4 (709) 15.9 (360)	22.4 (207) 28.3 (262) 33.7 (312) 15.7 (145)	24.9 (209) 30.3 (255) 30.3 (255) 14.5 (122)	0.063	27.0 (133) 25.4 (125) 28.8 (142) 18.9 (93)	0.161
Adverse health effects will occur because of FDNPP accident	Yes Probably Yes Probably no No	23.2 (524) 30.1 (680) 34.5 (780) 12.2 (276)	21.8 (202) 32.5 (301) 32.8 (304) 12.9 (119)	23.8 (200) 28.2 (237) 35.6 (299) 12.5 (105)	0.304	24.7 (122) 28.8 (142) 35.9 (177) 10.5 (52)	0.606
Genetic effects will occur because of the FDNPP accident	Yes Probably Yes Probably no No	18.5 (418) 29.7 (672) 38.2 (863) 13.6 (307)	16.6 (154) 31.5 (292) 37.3 (345) 14.6 (135)	19.5 (164) 29.4 (247) 38.5 (324) 12.6 (106)	0.775	20.3 (100) 27.0 (133) 39.4 (194) 13.4 (66)	0.850
Considered consulting a professional in regard to radiation	Yes No	11.1 (250) 88.9 (2010)	12.2 (113) 87.8 (813)	11.4 (96) 88.6 (743)	0.658	8.3 (41) 91.7 (452)	0.078
Motivated to learn more basic knowledge about radiation	Yes Probably Yes Probably no No	18.3 (413) 38.2 (864) 31.5 (711) 12.0 (272)	19.5 (181) 40.6 (376) 29.3 (271) 10.6 (98)	18.1 (152) 39.6 (333) 32.2 (271) 10.1 (85)	0.309	16.2 (80) 31.4 (155) 34.3 (169) 18.1 (89)	<0.001
Motivated to learn more about tritiated water	Yes Probably Yes Probably no No	29.9 (676) 37.4 (845) 23.1 (521) 9.6 (218)	31.3 (290) 36.6 (339) 23.5 (218) 8.5 (79)	31.3 (263) 40.8 (343) 20.3 (171) 7.6 (64)	0.062	24.9 (123) 33.1 (163) 26.8 (132) 15.2 (75)	<0.001
Have knowledge of a free food inspection center in Tomioka	Yes No	65.3 (1476) 34.7 (784)	74.4 (689) 25.6 (237)	63.4 (533) 36.6 (308)	<0.001	51.5 (254) 48.5 (239)	<0.001
Have knowledge of a free personal dosimeter rental system for residents	Yes No	51.3 (1159) 48.7 (1101)	55.5 (514) 44.5 (412)	50.5 (425) 49.5 (416)	0.040	44.6 (220) 55.4 (273)	<0.001
Have knowledge of a place that offers consultations about radiation	Yes No	38.9 (879) 61.1 (1381)	41.9 (388) 58.1 (538)	39.2 (330) 60.8 (511)	0.265	32.7 (161) 67.3 (332)	0.003

Chi-square test. Note: The FOV to Tomioka town: G1; more than twice a year; G2; once a year; G3, no visits. FDNPP = Fukushima Daiichi Nuclear Power Plant.

^a^G1 vs G2.

^b^G1 + G2 vs G3.

On the other hand, compared with G1 + G2, G3 had a significantly lower percentage of females (G3 vs G1 + G2; 40.8 vs 50.1%, *P* < 0.001), those aged ≥60 years (G3 vs G1 + G2; 61.3 vs 71.6%, *P* < 0.001), those who lived inside Fukushima (G3 vs G1 + G2; 64.1 vs 84.5%, *P* < 0.001), those who had decided to return to Tomioka (G3 vs G1 + G2; 6.3 vs 12.8%, *P* < 0.001), those who were still undecided about returning to Tomioka (G3 vs G1 + G2; 18.6 vs 26.5%, *P* < 0.001), those who knew about the free food inspection center in Tomioka (G3 vs G1 + G2; 51.5 vs 69.2%, *P* < 0.001) and those who knew about the free personal dosimeter rental system for residents (G3 vs G1 + G2; 44.6 vs 53.1%, *P* < 0.001). Furthermore, compared with G1 + G2, G3 also had significantly lower percentages of those who were motivated to gain more basic knowledge about radiation (G3 vs G1 + G2; 47.6 vs 59.0, *P* < 0.001) and those who were motivated to learn about tritiated water (G3 vs G1 + G2; 58.0 vs 69.9, *P* < 0.001). No significant differences in employment, living with a child, reluctance to consume food from Tomioka, concern that adverse health effects and genetic effects would occur because of the FDNPP accident and considering consulting a professional in regard to radiation were found between G1 + G2 and G3.

**Fig. 2:**
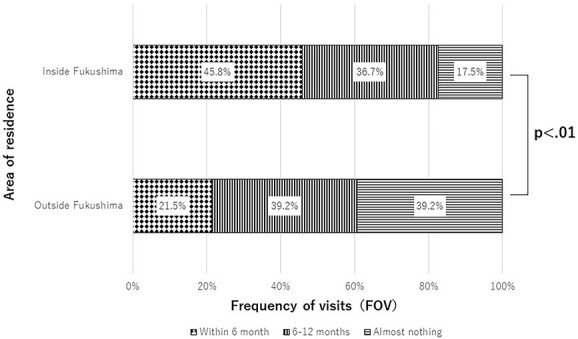
The FOV to Tomioka by the current area of residence. The vertical axis is the current area of residence and the horizontal axis is the FOV to Tomioka.

**Fig. 3:**
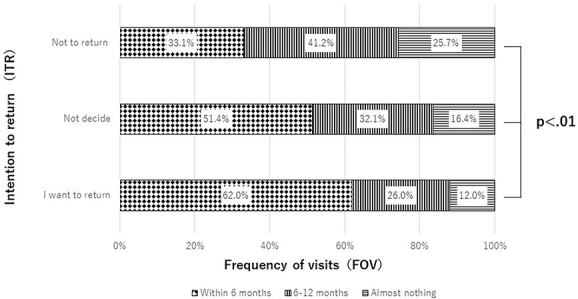
The FOV to Tomioka by ITR. The vertical axis is the ITR and the horizontal axis is the FOV to Tomioka.

In addition, we performed a further analysis in regard to the respondents’ current area of residence (Fig. 2), ITR (Fig. 3) and distribution of FOV to Tomioka in G1 to G3. In regard to areas of residence, the following significant differences were found between those who lived inside and outside of Fukushima: 45.8 vs 21.5% (G1), 36.7 vs 39.2% (G2) and 17.5 vs 39.2% (G3) (*P* < 0.01). Figure 3 shows the distribution of FOV for each ITR. The proportions of respondents in G1 who wanted to return, who were still undecided and who had decided not to return were 62.0, 51.4 and 33.1%, respectively. On the other hand, the proportions of those in G3 who wanted to return, who were still undecided and who had decided not to return were 12.0, 16.4 and 25.7%, respectively.

Table 2 shows the results of the multinomial regression analysis using G3 as a reference. Compared with G3, significantly higher percentages of residents in G1 (odds ratio [OR] = 5.4, 95% confidence interval [CI]: 4.1–7.3) and G2 (OR = 2.3, 95% CI: 1.8–3.0) lived inside Fukushima. Independent associations were also found between those aged ≥ 60 years in G1 (OR = 1.6, 95% CI: 1.3–2.1) and G2 (OR = 1.7, 95% CI: 1.3–2.1) compared with G3. Compared with G3, only G1 had a significantly higher percentage of residents who wanted to return to Tomioka (OR = 3.5, 95% CI: 2.3–5.4), were undecided about returning (OR = 2.5, 95% CI: 1.9–3.3), who were female (OR = 2.0, 95% CI: 1.6–2.6), were motivated to gain more basic knowledge about radiation (OR = 1.5, 95% CI: 1.2–2.1) and knew about a place for consultations on radiation (OR = 1.4, 95% CI: 1.1–1.8). The motivation to learn more about tritiated water was significantly higher in only G2 (OR = 1.8, 95% CI: 1.3–2.4) compared with G3.

**Table 2:** Results of the regression analysis with G3 as the reference

Variable	G3	G1	G2
	OR (Reference)	OR (95% CI)	OR (95% CI)
Current area of residence Inside/Outside of Fukushima	1	5.4^**^ (4.1–7.3)	2.3^**^ (1.8–3.0)
Age ≥60/<60	1	1.6^**^ (1.3–2.1)	1.7^**^ (1.3–2.1)
ITR I want to return/Others	1	3.5^**^ (2.3–5.4)	1.2 (0.8–2.0)
ITR Not decided yet/Others	1	2.5^**^ (1.9–3.3)	1.2 (0.9–1.6)
Sex Female/Male	1	2.0^**^ (1.6–2.6)	1.1 (0.9–1.4)
Motivation to know about tritiated water Yes/No	1	1.2 (0.9–1.6)	1.8^**^ (1.3–2.4)
Motivation to obtain more basic radiation knowledge Yes/No	1	1.5^**^ (1.2–2.1)	1.1 (0.8–1.5)
Recognize a place for consultations about radiation Yes/No	1	1.4^**^ (1.1–1.8)	1.2 (0.9–1.6)

Logistic regression analysis. OR = odds ratio and CI = confidence interval.

^*^ *P* < 0.05.

^**^ *P* < 0.01.

## DISCUSSION

In this study, we analyzed the FOV to Tomioka town and related factors among evacuees more than a decade after the FDNPP accident. The most interesting finding was the relationship between ITR and FOV among Tomioka residents: those who wanted to return visited Tomioka more often than those with other ITRs. Furthermore, those who were undecided regarding their ITR visited Tomioka more often than did those with other ITRs. These findings showed that residents who were interested in returning visited Tomioka more often. Regarding the decision to return, the residents of Tomioka required information about not only current radiation levels and the progress of decontamination in residential areas, but also about medical facilities, clinical departments, social welfare institutions, nursing homes and shopping malls [15]. Furthermore, the findings showed that the most important reasons for returning among those who had already returned were relaxation, peacefulness and familiarity with their hometown [24]. Therefore, it may be helpful to provide this type of information, including experiences of temporal life in areas where the evacuation order has been lifted, to residents trying to decide whether to return home.

Another fascinating result of this study was that ~70% of residents who had decided not to return had visited Tomioka once a year or more. A previous study reported that residents who had decided not to return to Tomioka had higher anxiety about radiation health effects for themselves and their children than did those with other ITRs [24]. Furthermore, these types of residents also had a higher perception of risk regarding the adverse effects of consuming food and drinking tap water from Tomioka than did those with other ITRs [16]. Surprisingly, no significant differences were found between these risk perceptions and the FOV. These results suggested that ITR, but not radiation risk perception, was associated with the FOV.

Nevertheless, the distribution of risk perceptions in this study was relatively high compared with previous studies [25, 26]. This study found that 52.7% of the respondents were reluctant to consume food from Tomioka, 53.3% were concerned about adverse health effects and 48.2% thought genetic effects would occur because of the FDNPP accident. On the other hand, the Fukushima Health Management Survey reported a decreased perception of risk of genetic effects (37.2%) in 2017 [27]. These results suggested that continuing risk communication with the residents of Tomioka is essential regardless of their FOV. Although the issue of genetic effects remains controversial, it is important to conduct risk communication from the perspective of human rights for victims of the Fukushima accident. A comparison between those who were living inside and outside of Fukushima revealed that the distance from Tomioka had a negative impact on their FOV. Those who lived inside Fukushima visited Tomioka more often than those who lived outside Fukushima. Fukushima is one of the largest prefectures in Japan, and so limited the contaminated areas immediately after the accident although most evacuees live outside Fukushima near the metropolitan cities such as Tokyo, which is >200 km from Tomioka [28]. One of the reasons for the residents not returning was because they had established a new life in a more convenient area. However, they also wanted to receive updated information about the progress of reconstruction in Tomioka and to maintain their relationship with their hometown, even though they had already decided not to return [24]. Although we found that the residents who lived farther from Tomioka had a lower FOV, additional research about factors related to the distance from Tomioka and their current living area, such as inside or outside of Fukushima, is needed.

G1, which had a higher FOV, possessed more widespread radiation-related information about Tomioka, such as knowledge of food inspection centers for internal exposure, the availability of a freely rentable personal dosimeter system for external exposure and places to consult about radiation.

After the FDNPP accident, Tomioka established a free personal dosimeter rental system, a food inspection center where anyone could measure radioactive substances free of charge and risk communication based on the results of measurements [19]. For effective radiation risk communication, it is essential to understand exposure doses and associated health risks [29]. Therefore, it is necessary to consider how to disseminate radiation-related information, including that about radiation doses and potential health effects, through newsletters, social networking, etc., to residents with a low FOV.

Finally, we asked about tritiated water from the FDNPP. The residents indicated wanting more knowledge about tritiated water than basic knowledge about radiation. The results of the multinomial logistic regression analysis showed that G2, who had a lower FOV than G1, wanted to know about tritiated water more than G3, who had no FOV. Tritiated water is treated water containing trace amounts of tritium that cannot be removed, even by using a special filter developed during the decommissioning process for the reactors at the FDNPP [17]. The FDNPP aims to release more tritiated water into the Pacific Ocean in the near future after fostering greater understanding among stakeholders and residents about the low radiation risk of health and environmental effects. The release of tritiated water is a serious issue that can damage the reputations of industries such as the fishing industry, as well as the reconstruction efforts in the FDNPP area, including Tomioka [18]. Although tritiated water is already being released from other nuclear power plants around the world, it is possible that the risk perception may differ between a planned release under normal operation and a release in response to a nuclear accident. The results of this study revealed that more residents wanted to learn about treated water than about basic radiation, regardless of their FOV. These findings suggest that after a nuclear accident, information on decommissioning should also be provided as part of risk communication.

This study had several limitations. First, we did not collect data on the reasons why the residents visited Tomioka or not. Defining the reasons for visiting would be useful information for recovery support and risk communication after a nuclear accident. Second, the residents in G3 may have had various reasons for not visiting Tomioka, even if they had wanted to, such as physical or mental disabilities. Third, the participants in this study were relatively old (about 70% were > 60 years of age), although the aging rate (number of those aged ≥ 65 years / the total number of residents) in Tomioka was ~30% [30]; this could have led to sampling bias. Despite these limitations, the results of this study revealed the FOV and related factors for previous residents of a town affected by a nuclear accident over a decade after the accident. After the FDNPP accident, many evacuees continued to visit Tomioka. These findings suggest the need to continue the effective dissemination of information about the effects of a nuclear accident and subsequent decommissioning process to evacuees after evacuation orders have been lifted.

## Data Availability

All data are available from the corresponding author upon reasonable request.
